# Anemia in Inflammatory Bowel Disease Outpatients: Prevalence, Risk Factors, and Etiology

**DOI:** 10.1155/2015/728925

**Published:** 2015-02-01

**Authors:** Carla Valéria de Alvarenga Antunes, Abrahão Elias Hallack Neto, Cristiano Rodrigo de Alvarenga Nascimento, Liliana Andrade Chebli, Ivana Lúcia Damásio Moutinho, Bruno do Valle Pinheiro, Maycon Moura Reboredo, Carla Malaguti, Antonio Carlos Santana Castro, Júlio Maria Fonseca Chebli

**Affiliations:** Division of Gastroenterology, Department of Medicine, Inflammatory Bowel Diseases Center, University Hospital of the Federal University of Juiz de Fora, University of Juiz de Fora School of Medicine, Avenida Eugênio do Nascimento s/n°, Dom Bosco, 36038-330 Juiz de Fora, MG, Brazil

## Abstract

Anemia is common in inflammatory bowel disease (IBD). However, epidemiological studies of nonwestern IBD populations are limited and may be confounded by demographic, socioeconomic, and disease-related influences. This study evaluated the prevalence, risk factors, and etiology of anemia in Brazilian outpatients with IBD. *Methods*. In this cross-sectional study, 100 Crohn's disease (CD) patients and 100 ulcerative colitis (UC) subjects were assessed. Anemia workup included complete blood count, ferritin, transferrin saturation, serum levels of folic acid and vitamin B_12_, and C-reactive protein (CRP) concentration. *Results*. The overall prevalence of anemia in IBD was 21%. There was no significant difference in the prevalence of anemia between CD subjects (24%) and UC (18%). Moderate disease activity (OR: 3.48, 95% CI, 1.95–9.64, *P* = 0.002) and elevated CRP levels (OR: 1.8, 95% CI, 1.04–3.11, *P* = 0.02) were independently associated with anemia. The most common etiologies of anemia found in both groups were iron deficiency anemia (IDA; 10% on CD and 6% on UC) followed by the anemia of chronic disease (ACD; 6% for both groups). *Conclusions*. In Brazilian IBD outpatients, anemia is highly concurrent condition. Disease moderate activity as well as increased CRP was strongly associated with comorbid anemia. IDA and/or ACD were the most common etiologies.

## 1. Introduction

Inflammatory bowel diseases (IBDs) are idiopathic multisystemic disorders that present with periods of relapses and remissions throughout their clinical course [1]. While ulcerative colitis (UC) is characterized by inflammation limited to the colonic mucosa, in Crohn's disease (CD) transmural inflammation is observed that can affect any part of the gastrointestinal tract [2]. In many patients, these conditions cause substantial personal cost, due to the unpredictable fluctuating symptoms, absenteeism at work, use of high-cost drugs, surgeries, or multidisciplinary care.

The severity of the intestinal manifestations (i.e., diarrhea, abdominal pain, and bleeding) is usually an important guide for management of IBD. Occasionally, however, one or more extraintestinal manifestations can be the predominant clinical feature on patients with IBD. Notably, anemia is one of the most common extraintestinal manifestations (or complications) in IBD [3–5]. In this clinical setting, anemia can contribute to patients' poor quality of life, particularly because of its negative impact on the feeling of wellbeing, physical performance, mood, cognitive function, and capacity to perform social activities [6, 7]. In addition, comorbid anemia in IBD individuals is a significant predictor of increased risk of hospitalization and even increased patient mortality [8–10]. Of note, anemia may occasionally both antecede the development of intestinal symptoms and be the key signal unmask IBD [3, 6].

The prevalence of anemia in IBD varies widely, with studies reporting a frequency between 8.8% and 74% depending on the assessed patient subpopulation [11, 12]. Etiology of anemia in IBD is multifactorial. Nonetheless, iron deficiency and characteristics of chronic disease are the most prevalent in this clinical setting [11–13].

Periodic screenings for anemia as well as a systematic diagnostic approach are essential steps to the appropriate management of this condition on IBD. However, anemia is both underdiagnosed and undertreated in populations with IBD [14]. Furthermore, different geographic, demographic, socioeconomic, and disease-related characteristics may exist between South American and North American or European populations that may limit the generalization of the findings regarding the epidemiology of anemia on IBD individuals, as the data are mainly derived from North American or European studies.

Therefore, the purpose of the present cross-sectional cohort study was to assess the prevalence, risk factors, and etiology of anemia in a Brazilian population with IBD. We hypothesized that anemia would be a concurrent condition in this patient population and is correlated with inflammatory activity.

## 2. Methods 

### 2.1. Study Design

We conducted a cross-sectional cohort study of adult outpatients with IBD from an IBD Center at the University Hospital of the Federal University of Juiz de Fora, Brazil, that were enrolled consecutively into this study for evaluation of the anemia between November 2012 and January 2013.

### 2.2. Participants

Our goal was to recruit 200 IBD patients (100 with CD and 100 with UC), nearly 20% of the IBD cohort followed in our Center.

The diagnosis of CD or UC was confirmed by combinations of clinical, radiologic, endoscopic, and histopathological criteria generally accepted for CD or UC [2]. The inclusion criteria were age ≥18 and <65 years and a confirmed diagnosis of IBD (CD or UC). Patients were excluded if they were younger than 18 or older than 65 years or presented with a severe IBD requiring hospitalization. Patients were also considered ineligible if they had a previous or current history of malignancies (except cutaneous), prior gastrectomy, systemic infections in the last 3 months, alcohol abusive use (daily alcohol consumption above 40 g), drug addiction, disabling chronic organ failure, or replacement therapy with iron, folic acid, or vitamin B_12_ in the last six months. Pregnant women or nursing mothers were not selected.

### 2.3. Measurements and Outcomes

#### 2.3.1. Sociodemographic and Disease-Related Characteristics

On inclusion, the eligibility criteria were assessed and medical history was recorded. Patient's relevant sociodemographic data included age, gender, current smoking (more than one cigarette daily), and history of alcohol consumption.

The classification and extent of the patients' IBD were established using the Montreal classification [15]. CD activity was measured according to Crohn's disease activity index (CDAI). Scores below 150 indicated remission and higher scores indicated active disease (i.e., mild to moderate disease: CDAI between 150 and 219; moderate to severe disease: CDAI between 220 and 450). For UC patients we used Truelove and Witt's criteria [16]. Thus, clinical remission was defined by ≤2 or 3 stools/day, without the presence of blood and/or pus in the stools, with no systemic symptoms; mild activity: up to 4 stools/day, with or without blood, no systemic involvement, and increased inflammatory markers; moderate activity: >4 stools per day with minimal systemic symptoms and increased inflammatory markers; severe activity: >6 stools per day with blood and evidence of systemic involvement, such as fever, tachycardia, anemia, and erythrocyte sedimentation above 30. In addition, disease duration and IBD-related surgical history were recorded as well.

#### 2.3.2. Evaluation of Anemia

During the inclusion in the study, blood samples (20 mL) for hematological and clinical chemistry were obtained. Anemia workup included complete blood count, ferritin, serum iron, transferrin saturation (TfS), serum levels of folic acid and vitamin B_12_, a reticulocyte count, erythrocyte sedimentation, and quantitative C-reactive protein (CRP) concentration.

According to the definition of the World Health Organization, the cutoff point for anemia was hemoglobin (Hb) levels below 13 g/dL in males and below 12 g/dL in nonpregnant females [17]. Severe anemia was defined as Hb < 10 g/dL for both genders. CRP was considered as elevated when the CRP levels were >6 mg/L. Patients were classified to have iron deficiency anemia (IDA) if there was a reduction in serum ferritin below 30 *μ*g/L or TfS was <16% in the absence of clinical and/or biochemical (i.e., CDAI ≤ 150 and CRP below or equal to upper limit of normality) data suggestive of disease activity; in the presence of inflammation (e.g., CDAI > 150 and/or CRP above upper limit of normality), IDA was diagnosed by a serum ferritin < 100 *μ*g/L and/or TfS < 16% [6].

In the presence of clinical or biochemical evidence of intestinal inflammation, the diagnostic criteria for anemia of chronic disease (ACD) were a serum ferritin > 100 *μ*g/L and TfS < 16% [6, 9]. A combination of IDA and ACD was defined by finding of a serum ferritin level between 30 and 100 *μ*g/L [6, 11]. Anemia due to vitamin B_12_ deficiency or anemia caused by folic acid deficiency was diagnosed when there were low serum levels of these vitamins in patients with anemia. If an absolute reticulocyte count was above 100,000, anemia was classified as hemolytic.

### 2.4. Statistical Analysis

The statistical analysis was performed using SPSS 16.0 (SPSS, Chicago, IL, USA). Continuous variables are presented as medians and ranges, and categorical variables are expressed as number and percentage of patients. Descriptive statistics of all relevant variables for the groups were calculated. For data analysis, the patients were divided into two groups (subjects with and without anemia) according to their Hb levels. Comparisons between groups as well as possible relationships between the presence of anemia with sociodemographic and disease-related data were analyzed using parametric Student's *t*-tests, nonparametric chi-squared tests, or the Mann-Whitney *U* test, as appropriate. Univariate and multivariate logistic analysis was performed to identify independent predictors for occurrence of anemia in the whole IBD group. The results are presented as odds risk (OR) and 95% confidence intervals (CI). For comparison, the level of statistical significance was set at *P* < 0.05 and all reported *P* values are two-tailed.

## 3. Results

### 3.1. Baseline Patient Characteristics

A total of 221 adult IBD outpatients were screened for the study. Of them, 18 individuals (8.1%) were not enrolled because they presented with some of the exclusion criteria: severe IBD requiring hospitalization (*n* = 4), colon cancer (*n* = 1), active infectious (*n* = 2), alcohol abusive use (*n* = 2), previous gastrectomy (*n* = 1), end-stage renal disease (*n* = 1), pregnancy (*n* = 2), and recent therapy with iron (*n* = 5). Three patients refused to participate after reading the informed consent. Thus 200 patients (90.5%) of the eligible population (75 males, 125 females, median age: 42 years (range 19–60)) were included. The demographic and clinical characteristics of the IBD cohort at baseline are shown in Table 1.

### 3.2. Assessment of Anemia in Patients with IBD

The overall prevalence of anemia was 21%. The prevalence was similar among patients with CD (24%) and UC subjects (18%; *P* = 0.25) (Table 1). No patient presented with severe anemia. There was no correlation between the occurrence of anemia with demographics, IBD location or duration, behavior of CD, and previous surgery for IBD. Conversely, IBD patients with moderate disease activity (CDAI between 220 and 450 for CD or >4 stools per day and increased inflammatory markers for UC) had significantly higher prevalence of anemia (66.7%) than patients in remission (17.8%) or with mildly active IBD (18.9%) (*P* < 0.001 for both comparisons). This finding was more notable for UC individuals with moderate disease activity (*P* = 0.001) than the CD patients (*P* = 0.024) presenting moderate activity (Table 1). In addition, CRP levels were significantly higher in both diagnostic groups with anemia than those in the population without anemia (*P* < 0.001 for CD and *P* = 0.009 for UC). Interestingly, smokers had a lower prevalence of anemia (*P* = 0.047).

As shown by univariate logistic regression analysis, the risk of anemia was 4-fold higher for moderate disease activity and 2-fold higher for no smoking (Table 2). Increase in CRP by each 1 mg/L increased this risk by 90%. In multivariate model, independent variables associated with anemia were moderate disease activity and CRP. Moderate disease increased 3.5-fold the risk of anemia, whereas each 1 mg/L increase in CRP increased this probability by 80%. However, in multivariate analysis, no smoking was not associated with an increased OR for anemia.

The most common etiologies of anemia found in both patients with CD and UC were IDA (10% in CD and 6% in UC subjects) followed by the ACD (6% in both groups) and B_12_ vitamin deficiency (5% in both groups). A total of 4% of the IBD cohort presented with concomitant IDA and ACD and only one patient showed folate deficiency associated with IDA (Table 3).

## 4. Discussion

This study demonstrated the magnitude of anemia in Brazilian patients with IBD. It clearly shows that current anemia was strongly associated with both moderate disease activity and higher CRP levels. To the best of our knowledge, this is the first study to demonstrate that moderate disease activity is a more important risk factor for anemia in UC patients than that in CD individuals. Furthermore, IDA and/or CDA were the most common etiologies of the anemia on the IBD setting.

The prevalence of anemia in our patients with IBD was 21%. This finding is in accordance with an overall prevalence of anemia (24%, 95% CI, 18–31) found on a recent meta-analysis evaluating 2192 IBD patients in European countries [18]. Other studies reported similar prevalence between 17% and 20% [19–21] which differs from the higher prevalence of anemia found in the studies of Bergamisch et al. [13] (65%) and Høivik et al. [4] (48.8% on CD and 20.2% on UC). This probably is due to the fact that our study was conducted in patients who were already under IBD treatment while the latter two studies evaluated patients before starting treatment. In current study, the prevalence of comorbid anemia was similar among patients with CD and UC subjects. Disparately, other studies reported anemia occurring more frequently in patients with CD than that in those with UC [4, 11]. It is possible that selection bias on inclusion in our study (i.e., more patients with UC presented with disease activity than CD patients) can justify this difference.

No patient in the current study had severe anemia. It, however, included only outpatients and individuals with severe IBD requiring hospitalization and higher probability of presenting with severe anemia were excluded. Furthermore, our study population is followed in an IBD center where patients are regularly monitored and treated to the target of tight control of intestinal inflammation. Notoriously, the degree of anemia in IBD patients correlates with underlying disease activity [18, 22]. It is another good reason to opt for expeditious, aggressive restraint of intestinal inflammation.

Similar to the finding in other studies [6, 7, 12, 19, 21], we have revealed that the two most commonly found patterns of anemia in IBD were IDA and that of chronic disease. Although the distinction between IDA and the ACD is important for IBD individuals, both conditions commonly overlap. It is well known that continuous or recurrent intestinal blood loss, reduced iron absorption from the inflamed bowel, and systemic iron sequestration drive a negative iron balance in IBD [1, 3, 11]. Conversely, a main mechanism driving ACD is the interleukins-6 release from the inflamed intestine that can trigger increased hepcidin hepatic synthesis, which potentially decreased duodenal absorption of iron and retained iron within cells of the reticular-endothelial system [23]. In addition, IBD individuals can have difficulty in utilizing iron appropriately because it has low levels of erythropoietin for their severity of anemia [22]. Systemic inflammatory cytokines increase (e.g., interleukins-6 and TNF-alpha, among others) has been shown to decrease mRNA expression of erythropoietin [24]. It is interesting to highlight that treatment with antitumor necrosis factor-alpha agents has been shown to improve iron deficiency by improving erythropoiesis, implicating a role for TNF-alpha in development of ACD and/or IDA in IBD patients [13].

A major finding of the present study was the strong correlation between anemia and disease activity in IBD patients. Moderate disease activity was an independent factor that increased 3.5-fold the risk of anemia. In particular, patients with moderate activity have a higher prevalence of anemia than those in remission or presenting with mild activity. This is the first study to show that this finding was more evident for UC subjects with moderate disease activity than the CD patients. It is possible to speculate that UC patients during flares can present with higher bleeding rate than CD patients, which can entail more IDA. This result suggests that, during follow-up of IBD patients, more intensive screening for anemia should be carried out during flare IBD, especially in UC patients with activity disease.

Other previous studies found that IBD patients with active disease status are more likely to have anemia than those being in remission [1, 11, 18]. Indeed, it should be emphasized that more inflammatory activity probably results in more blood loss, increased release of hepcidin, and decreased iron absorption from the intestine. Additionally, our observation that elevated CRP is positively associated with anemia is consistent with that of Høivik et al. [4]. It is worth remembering that CRP is an acute phase protein that represents a nonspecific serum marker of inflammation. Active IBD, especially CD, is associated with a CRP response in 40–80% of patients [25]. Thus, intestinal inflammation plays an integral role in the development of anemia in IBD individuals. Particularly in the setting of active disease and/or an increased CRP level, the suspicion of underlying anemia should be raised, which should be looked for carefully. However, it is important to point that about 18% (Table 1) of the patients with IBD had anemia despite having no active disease. Therefore, regardless of whether the patient has IBD in remission, screening for anemia may be still worthwhile.

Interestingly, in agreement with Høivik et al. [4], we have revealed a negative correlation between smoking and anemia by univariate analysis. It has been shown that smokers can develop secondary polycythaemia caused by exposure to carbon monoxide by smoking [26]. Nonetheless, in our multivariate model smoking was not associated with reduced odds for anemia. Hence, the possible protective role of the cigarette against the development of the anemia in IBD remains open to debate.

Ultimately, we believe that attention about the prevalence, risk factors, and different etiologies of anemia in patients with IBD could allow those personalized, targeted therapies for patients to be correctly adjusted according to the patient's need, thus potentially offering an opportunity for improvement of health-related quality of life, working ability, and patient satisfaction.

A limitation of the study is the fact that the occurrence of the anemia in IBD should ideally be assessed in a longitudinal rather than a cross-sectional study design. Furthermore, the burden of anemia found in our IBD patients may not completely reflect the true prevalence of anemia in the general IBD population. In the present study, the patients with IBD were at an IBD center of a university hospital, which makes them more likely to be a more ill or troubled group of IBD patients than those who would be seen in a community setting. It is generally accepted that patients with more severe disease are more likely to experience anemia [22]. This might bias the results toward a higher prevalence than those in the IBD population in general. Thus, our results should be interpreted in the appropriate context. Ultimately, future research through longitudinal studies is needed to examine what the impact of the aggressive management of IBD on both the incidence and severity of the anemia in IBD patients is.

## 5. Conclusion

In Brazilian IBD outpatients, anemia is highly concurrent condition, deserving special attention. IDA and ACD are the most common types of anemia in this clinical setting. Our data underline that anemia in IBD is largely correlated with inflammatory activity. So adequate treatment should target both the proper correction of Hb levels and aggressive management of active IBD with close monitoring, which could afford the opportunity for giving a better patient's quality of life. The high prevalence of anemia in IBD patients together with the availability of effective treatment for this condition indicates that it is timely to recommend that periodic screening and appropriate management for anemia should be carried out routinely as part of quality of care improvement for all patients with IBD.

## Figures and Tables

**Table 1:** Demographic and clinical characteristics of the inflammatory bowel disease patients with and without anemia.

	Crohn's disease		Ulcerative colitis		Total	
	Anemia	No anemia	Anemia	No Anemia	Anemia	No anemia
Total *n* (%)	24 (24)	76 (76)	18 (18)	82 (82)	42 (21)	158 (79)
Gender *n* (%)						
Female	11 (17.2)	53 (82.8)	11 (18)	50 (82)	22 (17.6)	103 (82.4)
Male	13 (36.1)	23 (63.9)	07 (17.9)	32 (82.1)	20 (26.7)	55 (73.3)
Age, year (median/range)	38.9 (18–64)	42.2 (19–63)	41.1 (20–64)	45.6 (19–65)	41.1 (18–64)	43.9 (19–65)
Disease activity^∗^ *n* (%)						
In remission	17 (19.5)	70 (80.5)	7 (14.6)	41 (85.4)	24 (17.8)	111 (82.2)
Mild	4 (50)	4 (50)	6 (13.3)	39 (86.7)	10 (18.9)	43 (81.1)
Moderate	3 (60)	2 (40)	5 (71.4)	2 (28.6)	8 (66.7)	4 (33.3)
CD location *n* (%)						
Ileal	8 (34.8)	15 (65.2)				
Colonic	7 (20.6)	27 (79.4)				
Ileocolonic	9 (20.9)	34 (79.1)				
UC location *n* (%)						
Ulcerative proctitis			8 (15.1)	45 (84.9)		
Left sided UC			2 (10)	18 (90)		
Extensive UC			8 (29.6)	19 (70.4)		
Behavior of CD *n* (%)^†^						
B1	8 (21.6)	29 (78.4)				
B2	7 (25)	21 (75)				
B3	9 (25.7)	26 (74.3)				
Disease duration (year) (median/range)	6.4 (1.4–12.8)	8 (0.8–11.6)	7.4 ± 7.3 (2.7–10.8)	7.7 ± 5.2 (1–11)	6.8 ± 7.0 (1.4–12.8)	7.8 ± 6.2 (0.8–11.6)
Previous surgery *n* (%)						
Yes	9 (23.1)	30 (76.9)	1 (50)	1 (50)	10 (24.4)	31 (75.6)
No	15 (24.6)	46 (65.4)	17 (17.3)	81 (82.7)	32 (20.1)	127 (79.9)
Smoking^∗∗^ *n* (%)						
Yes	2 (11.1)	16 (88.9)	1 (5.9)	16 (94.1)	3 (8.6)	32 (91.4)
No	22 (26.8)	60 (73.2)	17 (20.5)	66 (79.5)	39 (23.6)	126 (76.4)
CRP^∗∗∗^ (mg/L) (median/range)	12.9 (1–80)	4.6 (1–45)	9.9 (1–60)	4.9 (1–30)	11 (1–80)	3.5 (1–45)

CD: Crohn's disease; UC: ulcerative colitis; CRP: C-reactive protein.

^∗^For moderate disease activity there was a statistically significant difference with *P* = 0.024 (CD), *P* = 0.001 (UC), and *P* < 0.001 (for total population).

^∗∗^For smoking there was a statistically significant difference with *P* = 0.047 (for total population).

^∗∗∗^For CRP there was a statistically significant difference with *P* < 0.001 (CD) and *P* = 0.009 (UC).

^#^For all other characteristics, there was no significant difference.

^†^B1: nonstricturing, nonpenetrating; B2: stricturing; B3: penetrating.

**Table 2:** Univariate and multivariate logistic regression analysis for occurrence of anemia in the whole inflammatory bowel disease group.

	OR	−95% CI	+95% CI	*P *
Univariate logistic regression analysis for anemia risk				
Gender	0.957	0.912	1.242	0.63
Age	1.263	0.674	3.487	0.59
Inactive disease	1.438	0.497	2.764	0.75
Mild disease activity	2.614	1.345	6.243	0.09
Moderate disease activity	4.101	2.503	10.542	<0.001
Disease duration	0.989	0.646	1.728	0.21
Previous surgery	1.345	0.587	3.843	0.57
No smoking	1.912	1.088	4.531	0.04
CRP	1.905	1.047	3.354	0.001

Multivariate logistic regression analysis for anemia risk				
No smoking	0.912	0.878	2.531	0.14
Moderate disease activity	3.487	1.954	9.643	0.002
CRP	1.803	1.048	3.113	0.02

OR: odds risk; CI: confidence intervals; CRP: C-reactive protein.

**Table 3:** Etiology of the anemia on patients with inflammatory bowel disease.

	ACD	B_12_ deficiency	IDA	ACD + IDA
	(%)	(%)	(%)	(%)
Crohn's disease (*n* = 100)	6	5	10^∗^	3
Ulcerative colitis (*n* = 100)	6	5	6	1

Total (*n* = 200)	12	10	16	4

ACD: anemia of chronic disease; IDA: iron deficiency anemia.

^∗^One Crohn's disease patient presented with concomitant IDA and folate deficiency.
